# Diagnostic performance between in-house and commercial SARS-CoV-2 serological immunoassays including binding-specific antibody and surrogate virus neutralization test (sVNT)

**DOI:** 10.1038/s41598-022-26202-1

**Published:** 2023-01-02

**Authors:** Poramed Winichakoon, Jiraprapa Wipasa, Kriangkrai Chawansuntati, Parichat Salee, Tavitiya Sudjaritruk, Saowaluck Yasri, Chantana khamwan, Rungnapa Peerakam, Dararat Dankai, Romanee Chaiwarith

**Affiliations:** 1 Division of Infectious Diseases and Tropical Medicine, Department of Internal Medicine, Faculty of Medicine, Chiang Mai University, Chiang Mai, Thailand; 2 Research Institute for Health Sciences, Chiang Mai University, Chiang Mai, Thailand; 3 Division of Infectious Diseases, Department of Pediatrics, Faculty of Medicine, Chiang Mai University, Chiang Mai, Thailand; 4 Diagnostic Laboratory, Maharaj Nakorn Chiang Mai Hospital, Faculty of Medicine, Chiang Mai University, Chiang Mai, Thailand

**Keywords:** Diagnosis, Medical research

## Abstract

This study aimed to evaluate the correlation between in-house and commercial binding-specific IgG antibodies and between in-house and commercial SARS-CoV-2 surrogate virus neutralization tests (sVNT). Samples from healthcare workers who received vaccines against SARS-CoV-2 were tested for RBD-specific antibody, S-specific antibody, and in-house ELISA, commercial sVNT, and in-house sVNT, against wild-type SARS-CoV-2. Three hundred and five samples were included in the analysis. The correlation between S-specific binding antibodies and in-house ELISA was 0.96 (95% CI 0.96–0.97) and between RBD-specific antibodies and in-house ELISA was 0.96 (95% CI 0.95–0.97). The Cohen’s kappa between in-house sVNT and the commercial test was 0.90 (95% CI 0.80, 1.00). If using 90% inhibition of sVNT as the reference standard, the optimal cut-off value of RBD-specific antibodies was 442.7 BAU/mL, the kappa, sensitivity, and specificity being 0.99, 99%, and 100%, respectively. The optimal cut-off value of S-specific antibodies was 1155.9 BAU/mL, the kappa, sensitivity, and specificity being 0.99, 100%, and 99%, respectively. This study demonstrated a very strong correlation between in-house ELISA and 2 commercial assays. There was also a very strong correlation between in-house and commercial SARS-CoV-2 sVNT, a finding of particular interest which will inform future research.

## Introduction

To date, 4 different platforms of SARS-CoV-2 vaccine were labelled as Emergency Use Authorization by WHO, specifically, mRNA vaccines, viral-vector vaccines, protein subunit vaccines, and whole-cell inactivated virus vaccines^1^. Those vaccines have shown immunogenicity, efficacy, and effectiveness against SARS-CoV-2^2–5^. Vaccines elicit humoral and cellular immune responses which work together to protect against infection, in this case SARS-CoV-2 infection^6,7^. It has been postulated that while the neutralizing antibodies are essential for controlling SARS-CoV-2 infection, an effective clearance of the virus is dependent on T cells^8–11^. However, there is some evidence to suggest that cell-mediated immunity is able to control COVID-19 without substantial contribution from neutralizing antibodies^12–15^. Most vaccines including some COVID-19 vaccines are believed to protect against disease through neutralizing antibodies^2,7,16–18^.

Due to technical and expertise requirements for determining cell-mediated immune response, detection of humoral response is more achievable. The humoral immune response can be measured by the detection of either: (1) binding antibodies i.e. immunoglobulin G (IgG) level against anti-S (spike protein), anti-receptor-binding domain (anti-RBD) on S1 subunit or (2) neutralizing antibody detection tests i.e. the gold-standard manual plaque reduction neutralization test (PRNT), pseudotyped virus neutralization assay (PNA), or surrogate virus neutralization test (sVNT)^19–21^. The neutralizing antibody determines the efficacy of performance of an antibody to prevent infection by SARS-CoV-2 in vitro. Currently, there is no recommendation for generalized using of antibody testing to assess for immunity against SARS-CoV-2 after COVID-19 vaccination, to determine the necessity for vaccination in an unvaccinated individual or requirement for booster dose^22^. However, antibody testing may be applied for occupational health, public health, and research purposes^22^. Neutralizing antibody detection tends to be an acceptable predictor and may be used as a key marker for vaccine efficacy during clinical trials^23^, and a correlation has been shown between the level of antibody and protection against symptomatic COVID-19 disease^24,25^ Due to the high cost of available commercial kits, we developed an in-house enzyme-linked immunosorbent assay (ELISA) for detection of anti-S-IgG antibody and in-house neutralizing antibody test kit (sVNT) for current research purposes.

Thus, this study was conducted to: (1) evaluate the correlation among binding antibody assays i.e. an in-house ELISA and two commercial immunoassays; the Elecsys anti-SARS-CoV-2 S immunoassay (Roche Diagnostics) [S-specific-IgG-test] and the SARS-CoV-2 IgG II Quant assay (Abbott Laboratories) [RBD-specific-IgG-test] in serum from vaccinated adults; (2) evaluate the correlation between neutralizing antibody tests i.e. the SARS-CoV-2 NeutraLISA assay (EUROIMMUN) [commercial sVNT] and the in-house SARS-CoV-2 surrogate virus neutralization test [in-house sVNT], and (3) identify the level of binding specific IgG antibody which was correlated with % inhibition of commercial sVNT.

## Results

Out of 153 participants who previously received 2 doses of CoronaVac® vaccine, 39 patients (25.5%) were male and the median age was 44 (IQR 30, 53) years. There were 305 specimens consisting of 77 samples at before the third dose of ChAdOx1 nCoV-19 vaccine, 76 samples at before the third dose of the BNT162b2 mRNA vaccine, 76 samples at 4 weeks after the third dose of ChAdOx1 nCoV-19 vaccine, and 76 samples at 4 weeks after the third dose of BNT162b2 mRNA vaccine. A total of 305 specimens were analysed.

The median (IQR) RBD-specific antibody, S-specific binding antibody, and in-house S-specific binding antibody before the third dose of vaccine were 65.6 (36.1, 101.2), 62.3 (32.9, 118.5) and 18.5 (10.8, 28.1) BAU/mL, respectively. They were 1821.4 (1096.7, 2892.8), 9213.2 (5022.5, 15330.6), and 467.2 (392.1, 539.4) BAU/mL at 4 weeks after the third dose of vaccine, respectively. The median % (IQR) inhibitions of commercial sVNT and in-house sVNT were 28.2 (13.3, 45.9) % and 22.8 (15.9, 39.8) %, respectively before the third dose and 99.5 (99.1, 99.7) % and 98.5 (98.2, 98.6) % at 4 weeks after the third dose, respectively.

### The correlations among binding-specific IgG antibody tests

**Figure 1:**
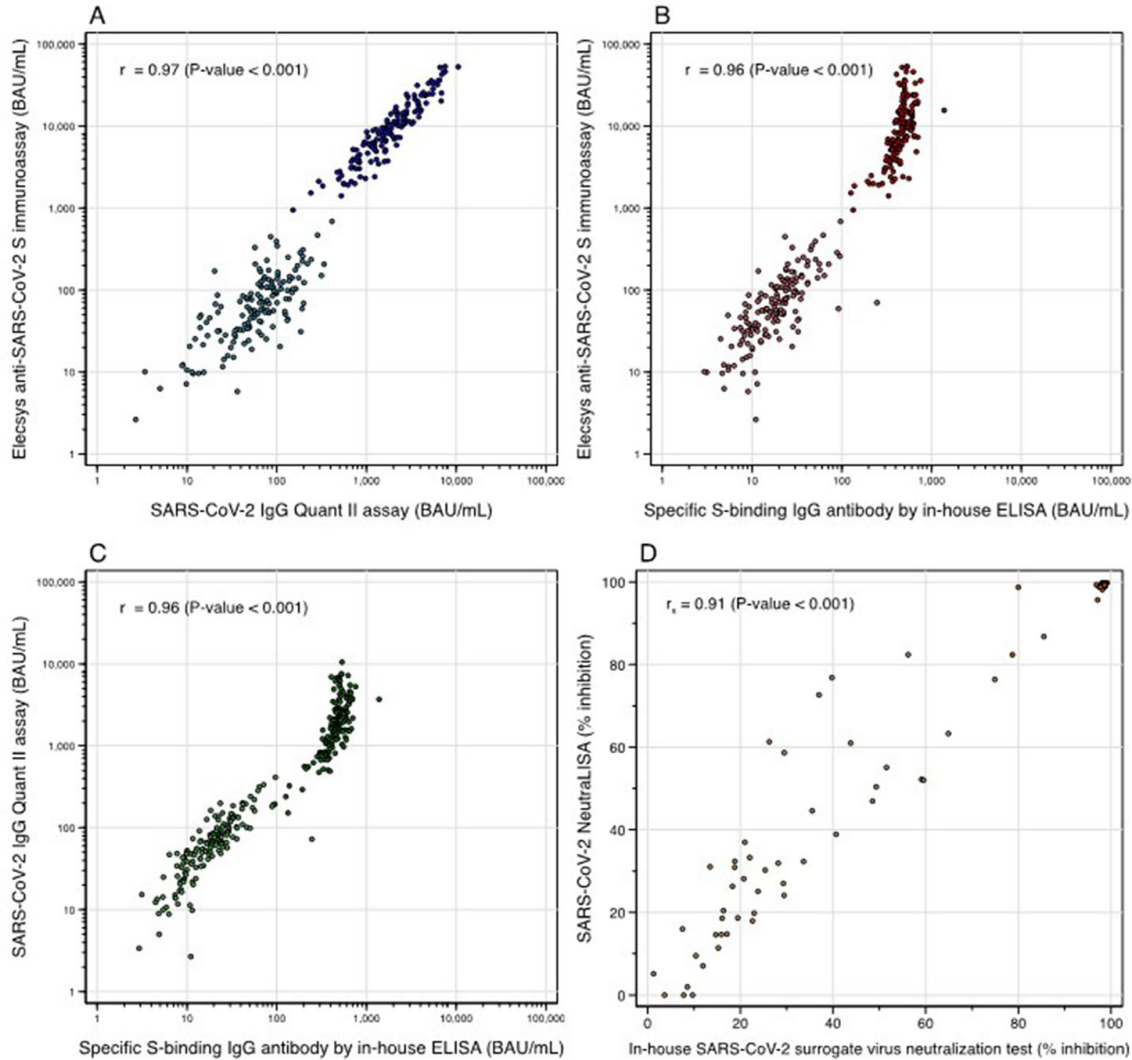
The correlations among binding-specific IgG antibody tests (**A**, **B**, **C**) and between in-house SARS-CoV-2 surrogate neutralization test and SARS-CoV-2 NeutraLISA (**D**).

The correlation in log 10 scale between the RBD-specific-IgG-test and S-specific IgG-test was 0.97 (95% CI 0.96–0.98) (Fig. 1A), between S-specific-IgG-test and in-house ELISA was 0.96 (95% CI 0.96–0.97) (Fig. 1B), and between the RBD-specific-IgG-test and in-house ELISA was 0.96 (95% CI 0.95–0.97) (Fig. 1C).

### The correlation between in-house sVNT and the commercial sVNT

The correlation of % inhibition between in-house sVNT and the commercial sVNT was 0.91 (95% CI 0.87–0.94, *p*-value < 0.001) (Fig. 1D). Converting from in-house sVNT to the commercial sVNT, the equation used was: % inhibition from in-house sVNT = (0.9779 × % inhibition from the commercial sVNT) + 1.1068. Another way, an alternative calculation was % inhibition from the commercial sVNT = (0. 9968 x % inhibition from in-house sVNT) + 1.3587.

### The optimal cut-off value of binding specific IgG antibody using the commercial sVNT as a reference standard

- At a cut-off level of 35% inhibition of commercial sVNT

RBD-specific-IgG-test which designated 7.1 BAU/mL as a cut-off level of positive result, had 69% agreement with Cohen's kappa 0.04, 100% sensitivity, and 3% specificity. The S-specific-IgG-test, which designated 0.8 BAU/mL as a cut-off level of a positive result, also had a 68% agreement with kappa 0, and 100% sensitivity.

**Figure 2:**
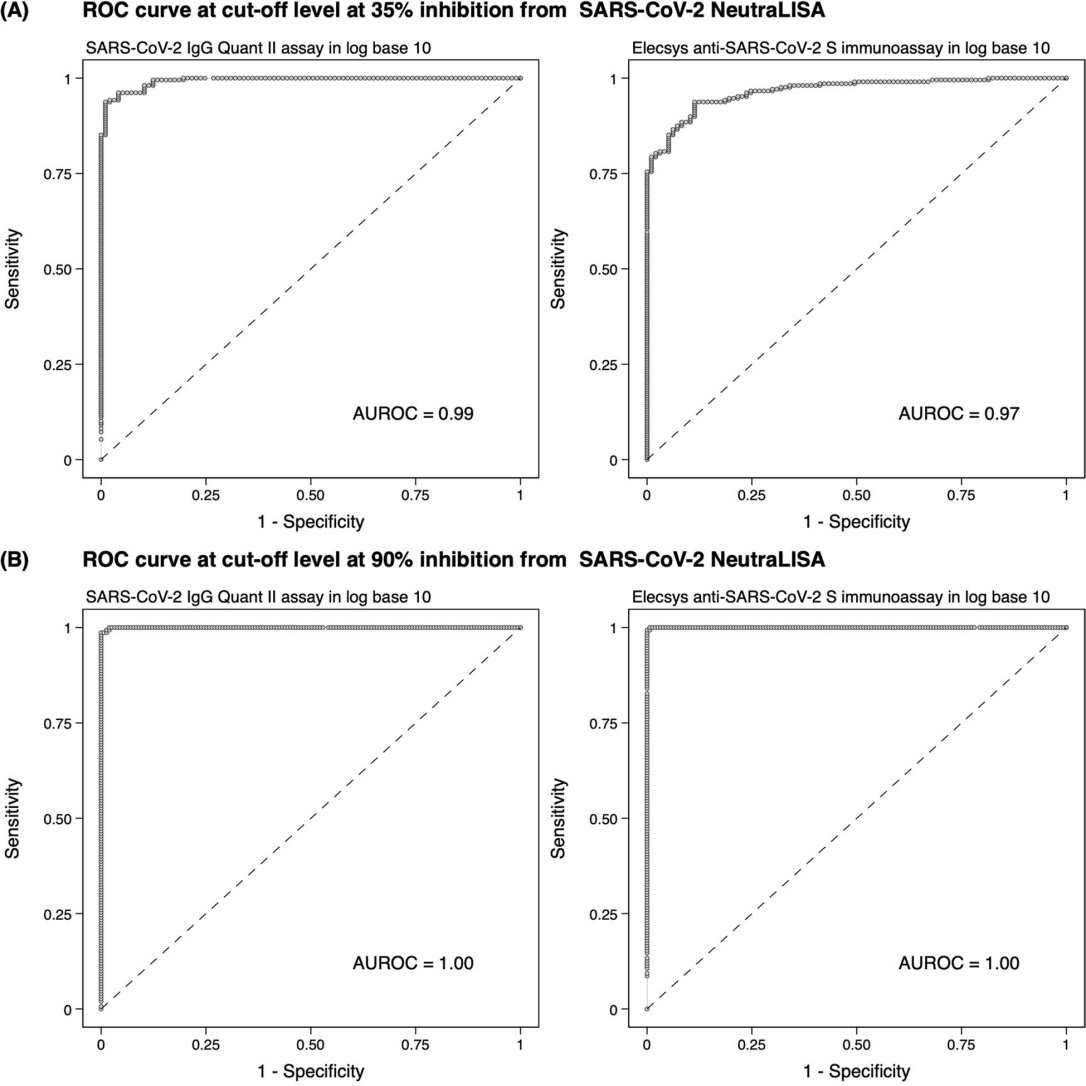
The receiver operating characteristic (ROC) curve analysis of binding-specific antibody at 35% (**A**) and 90% (**B**) inhibition from SARS-CoV-2 NeutraLISA.

After 4 sequential steps were applied, the area under the ROC (AUROC) curve of RBD-specific-IgG-test and S-specific-IgG-test was 0.99 and 0.97, respectively. (Fig. 2A) The cut-off value for RBD-specific-IgG-test was 83.9 BAU/mL, which increased the agreement to 96%, with kappa 0.91. The sensitivity and the specificity were 96% and 96%, respectively. The optimal cut-off value for S-specific-IgG-test was 90.9 BAU/mL. By using this cut point, the agreement increased to 91% with kappa 0.82. The sensitivity and specificity were 94% and 89%, respectively. (Tables 1 and 2).

- At a cut-off level of 80%, 85%, 90%, and 95% inhibition of commercial sVNT

**Table 1:** The optimal cut-off value of SARS-CoV-2 Quant II using the SARS-CoV-2 NeutraLISA as a reference standard.

SARS-CoV-2 Quant II (BAU/mL)	NAb (% inhibition)		% Agreement	Kappa (95% CI)	*p*-value	Sensitivity (95% CI)	Specificity (95% CI)
	< 35	≥ 35	96.1	0.91 (0.86, 0.96)	< 0.001	0.96 (0.93, 0.98)	0.96 (0.90, 0.99)
< 83.9	93	8					
≥ 83.9	4	200					
	< 80	≥ 80	98.4	0.97 (0.94, 1)	< 0.001	0.99 (0.95, 1)	0.98 (0.94,1)
< 210.8	146	2					
≥ 210.8	3	154					
	< 85	≥ 85	99.3	0.99 (0.97, 1)	< 0.001	1.00 (0.98, 1)	0.99 (0.95, 1)
< 261.9	151	0					
≥ 261.9	2	152					
	< 90	≥ 90	99.3	0.99 (0.97, 1)	< 0.001	0.99 (0.95, 1)	1.00 (0.98, 1)
< 442.7	155	2					
≥ 442.7	0	148					
	< 95	≥ 95	99.7	0.99 (0.98, 1)	< 0.001	0.99 (0.96, 1)	1.00 (0.98, 1)
< 442.7	156	1					
≥ 442.7	0	148

**Table 2:** The optimal cut-off value of the Elecsys anti-SARS CoV-2 immunoassay using the SARS-CoV-2 NeutraLISA as a reference standard.

Elecsys (BAU/mL)	NAb (% inhibition)		% Agreement	Kappa (95%CI)	*p*-value	Sensitivity (95% CI)	Specificity (95% CI)
	< 35	≥ 35	91.2	0.82 (0.75, 0.89)	< 0.001	0.94 (0.90, 0.97)	0.89 (0.81, 0.94)
< 90.9	86	13					
≥ 90.9	11	195					
	< 80	≥ 80	98.7	0.97 (0.95, 1)	< 0.001	0.98 (0.95, 1)	0.99 (0.96, 1)
< 457.8	148	3					
≥ 457.8	1	153					
	< 85	≥ 85	99.0	0.98 (0.96, 1)	< 0.001	0.99 (0.95, 1)	0.99 (0.96, 1)
< 1155.9	152	2					
≥ 1155.9	1	150					
	< 90	≥ 90	99.7	0.99 (0.98, 1)	< 0.001	1.00 (0.98, 1)	0.99 (0.97, 1)
< 1155.9	154	0					
≥ 1155.9	1	150					
	< 95	≥ 95	99.7	0.99 (0.98, 1)	< 0.001	0.99 (0.96, 1)	1.00 (0.98, 1)
< 1888.1	156	1					
≥ 1888.1	0	148					

The description was based on the example of 90% inhibition. RBD-specific-IgG-test which designated 7.1 BAU/mL as a cut-off level of a positive result, had a 50% agreement with kappa 0.02 (95% CI 0–0.04), 100% sensitivity (95% CI 98–100), and 2% specificity (95% CI 0–6), The S-specific-IgG-test, which designated 0.8 BAU/mL as a cut-off level of a positive result, also had a 49% agreement with kappa 0, sensitivity 100%.

After 4 sequential steps were applied, the AUROC curve of RBD-specific-IgG-test and S-specific-IgG-test were 1 and 1, respectively. (Fig. 2B) RBD-specific-IgG-test had 99% agreement with kappa 0.99 at an optimal cut-off value of 442.7 BAU/ml with 99% sensitivity, and 100% specificity. At an optimal cut-off value of 1155.9 BAU/mL, S-specific-IgG-test had 100% agreement with kappa 0.99. The sensitivity of this assay was 100% and the specificity was 99%. The optimal cut-off value, % agreement, kappa, sensitivity and specificity for 80%, 85%, and 95% inhibition are shown in Tables 1 and 2.

### The kappa between in-house SARS-CoV-2 sVNT and the commercial sVNT

At the cut-off level of 35% inhibition for the commercial sVNT and 30% for in-house sVNT, the agreement was 95.7%, and the kappa was 0.90 (95% CI 0.80, 1.00). Using the commercial sVNT as a reference standard, the sensitivity and the specificity of in-house sVNT was 95% (95% CI 87–99%), and 97% (95% CI 82–100%), respectively.

## Discussion

This study demonstrated a “very strong” correlation among SARS-CoV-2 IgG Quant II assay, Elecsys anti-SARS-CoV-2 S immunoassay, and S-specific antibodies by in-house ELISA, with a correlation above 0.95. Several studies have reported moderate to strong correlations between commercially available immunoassays^26–28^. Although the correlation was very strong as regards direction, S-specific antibody by Elecsys anti-SARS-CoV-2 S immunoassay had a higher level than RBD-specific antibody detected by SARS-CoV-2 IgG Quant II assay at 4-weeks after, but not before the third dose vaccination. This finding has also been reported by Perkmann T. et al., which found that the correlation between Elecsys anti-SARS-CoV-2 S immunoassay and Abbott Architect RBD-specific antibodies was different at different time points after vaccination^29^. This emphasizes the importance of detailed interpretation of the results, particularly from different tests and at different time points. In addition, the antibodies from the in-house ELISA had lower levels than both S-specific antibodies by Elecsys Anti-SARS-CoV-2 S immunoassay and RBD-specific antibodies. (Fig. 1) A comparison between commercial and in-house techniques which detect antibodies to the spike protein but by different methods i.e. ECLIA in the former and ELISA in the latter, the antibody levels from in-house ELISA were lower. The correlation was weaker at the time point after the third dose than before. Similar findings were found when comparing the antibodies from in-house ELISA with the RBD-specific antibodies. In the case of the neutralizing antibodies, the correlation between in-house sVNT and commercial sVNT was also “very strong” with a correlation of 0.91, although there were some scattered results away from the regression line at 30–70% inhibition. There were satisfactory results of the in-house ELISA and also in-house sVNT with the commercially available tests. These tests may be cost effective alternative ways of detecting antibodies with acceptable performance.

Several studies showed that vaccine efficacy and vaccine effectiveness correlated with neutralizing antibody levels for protection against symptomatic COVID-19 infection^9,24,30^. Protection against severe disease required lower neutralizing antibody levels than those conferring protection against mild disease^9^. Predictive models identifying a mean neutralization titer (relative to convalescent) of 1 correlate with 80% protective efficacy against SARS-CoV-2 infection, mostly due to SARS-CoV-2 variant B.1.177^24^. That study predicted vaccine efficacy against symptomatic infection of 50%, 60%, 70%, 80%, and 90% with the level of anti-spike IgG, anti-RBD IgG, normalized live-virus neutralization assay and pseudovirus neutralization assay respectively^24^. For example, 80% vaccine efficacy against symptomatic COVID-19 infection with mostly B.117 variants of SARS-CoV-2 was achieved with anti-spike IgG of 40,923 AU/mL, and 264 BAU/mL and anti-RBD-IgG of 63,383 AU/ml, and 506 BAU/mL, and NF50 for normalized live-virus neutralization assay of 247, and ID50 for pseudovirus neutralization assay of 57. No data to compare the vaccine efficacy and % inhibition of sVNT was published in that study. However, a correlation between sVNT and virus neutralization test (the plaque-reduction neutralization test; PRNT) has been reported^31^.

A cut-off level of positive result from SARS-CoV-2 IgG Quant assay and Elecsys anti-SARS-CoV-2 S immunoassay demonstrated no agreement with a cut-off level of seroconversion by commercial sVNT. After the 4 sequential steps were applied, the optimal cut-off values for SARS-CoV-2 IgG Quant assay and Elecsys anti-SARS-CoV-2 S immunoassay with 35% inhibition of commercial sVNT were 83.9 BAU/mL and 90.9 BAU/mL, respectively. The level of 442.7 BAU/mL for SARS-CoV-2 IgG Quant assay and 1155.9 BAU/mL showed almost perfect agreement with 90% inhibition. We also performed tests at the cut points of 80%, 85%, and 95% inhibition, and the results are shown in Tables 1 and 2. The sensitivity and specificity were not reduced above 85% inhibition. However, the results must be interpreted with caution as the assays were tested against wild-type SARS-CoV-2, but not their variants.

The kappa statistic between in-house sVNT and the commercial sVNT was almost perfect. We have proposed an equation for switching between them. However, this equation may only be suitable for this study population and for research purpose when the costs are limited.

With straightforward technical requirements and lower operating costs, the determination of binding-specific antibody levels was more convenient for evaluating the immune response after vaccination against SARS-CoV-2 infection or immunity after infection. Furthermore, when long-acting antibodies (LAAB) are available for use for pre-exposure prevention of COVID-19 in certain populations, the antibody level may help to guide selection of eligible patients. For example, the Department of Disease Control, Ministry of Public Health of Thailand suggested prescribing LAAB for patients with end-stage renal disease (ESRD), kidney or other organ-transplant or bone marrow transplant who received immunosuppressive therapy, ESRD on hemodialysis or peritoneal dialysis, who had received at least 3 doses of vaccine against SARS-CoV-2 but had anti-spike IgG of/ or comparable to < 264 BAU/mL, as a first priority due to an imbalance between certain population and available LAAB^32^.

This study has several limitations. First, the binding specific antibodies and % inhibition from sVNT were tested against wild-type SARS-CoV-2 virus. The major variants of interest (VOIs), and variant of concerns (VOCs) required higher levels of antibodies than against wild-type SARS-CoV-2^30^. Interpretation and the cut-off levels from this study may not be applied the current situation where circulating virus are VOIs and VOCs. Second, the reference standard used in this study was the sVNT, which was not the gold standard for neutralizing antibody detection. Although there was a correlation between sVNT and the PRNT, the results must be interpreted with caution. Third, although in-house sVNT and in-house ELISA provide satisfactory results and has economic advantages when compared to the commercial assays, they require an overnight incubation of ACE-2 or the S antigen in ELISA plates. Therefore, they take more time to complete the assays compared to the ready to use commercial kits.

In conclusion, this study demonstrated a very strong correlation between in-house ELISA with 2 commercially assays available in Thailand, i.e. the SARS-CoV-2 IgG II Quant assay and the Elecsys anti-SARS-CoV-2 S immunoassay. However, when testing for binding specific antibody, the same tests must be used for longitudinal study as the antibody levels in each test were different (Fig. 1) There was also a very strong correlation between in-house sVNT and the commercial sVNT which is of great interest and will inform future research. These findings are very helpful for both research purposes, and to save costs with acceptable levels of outcome.

## Methods

This cross-sectional study was conducted at Maharaj Nakorn Chiang Mai Hospital, a tertiary care hospital affiliated to Chiang Mai University from September to December 2021. For comparison of the tests, left-over serum samples taken from healthcare workers who enrolled onto a study into immunogenicity against SARS-CoV-2 after vaccination and had provided written informed consent for future studies were collected. In brief, participants were healthcare workers aged 18–60 years, previously received 2 doses of CoronaVac® vaccine, and received the third dose of vaccine either ChAdOx1 nCoV-19 (AZD1222) or BNT162b2 mRNA. Only serum from participants at before and 4 weeks after the third dose of vaccine were selected.

Serum was separated to test for: (1) S-specific-IgG-test, (2) RBD-specific-IgG-test, (3) in-house ELISA, (4) commercial sVNT, and (5) in-house sVNT. All tests were performed against wild-type SARS-CoV-2 virus.

## Laboratory assays

### Binding antibody

#### The SARS-CoV-2 IgG II Quant assay (Abbott Laboratories Inc, IL, USA)^33^

This an automated, two-step immunoassay was designated for the qualitative and semi-quantitative detection of IgG antibodies to SARS-CoV-2 in human serum and plasma using chemiluminescent microparticle immunoassay (CMIA) technique. The amount of antibodies were presented as arbitrary units (AU)/mL and this assay measured the concentration of anti-RBD-WT (wild-type) IgG levels between 21 and 40,000 AU/mL. Those values were converted to binding antibody units (BAU)/ mL by multiplying by 0.142 per WHO recommendations^34^. The cut-off level for a positive result was ≥ 50 AU/ml (7.1 BAU/mL).

#### The Elecsys Anti-SARS-CoV-2 S immunoassay (Roche Diagnostics International Ltd, Rotkreuz, Switzerland)^35^

Double-antigen sandwich is a major principle of the test. The reagent consists of antigens which predominantly captured anti‑SARS‑CoV‑2 IgG, but also captured anti‑SARS‑CoV‑2 IgA and IgM, levels being determined by electrochemiluminescence immunoassay (ECLIA). The values were presented as U/mL and this assay measured the concentration of anti-S-WT (wild-type) IgG levels between 0.4 and 250 U/mL. The samples with anti‑SARS‑CoV‑2‑S concentrations over the measurable range were diluted with Diluent Universal up to 1:100 dilution. Those values were converted to BAU/ mL by multiplying by 1.029 as per WHO recommendations (44). The cut-off level of a positive result was ≥ 0.8 U/mL (0.8232 ~ 0.8 BAU/mL)^34^.

#### Determination of specific S-binding IgG antibody by in-house ELISA

Antibodies specific to the spike protein were determined by indirect ELISA. Fifty microliters of 1 µg/mL spike proteins (Genscript, Piscataway, USA) in bicarbonate buffer (pH 9.6) were added to 96-well Maxisorp immunoplates (Thermo scientific, Roskilde, Denmark). After incubation overnight at 4 °C, plates were washed with washing buffer (0.05% Tween-20 (Calbiochem, Gibbstown, USA) in phosphate buffer saline (PBS) and blocked with 2% skimmed milk at 37 °C for 1 h. Fifty microliters of samples were diluted in the ratio 1:100 and serially diluted positive controls were added and incubated at 37 °C for 1 h. After washing, 50 µl of 1: 2000 goat anti-human IgG conjugated with horse radish peroxidase (HRP) (Invitrogen, Carlsbad, USA) were added and incubated at 37 °C for 1 h. After washing, 50 µl of tetramethylbenzidine (TMB) substrate (Life Technologies, Frederick, USA) were added and plates were incubated at room temperature for 30 min. The enzyme reaction was terminated using 0.2 M sulfuric acid and absorbance was read at 450 nm on a microplate reader (CLARIOstar®, Ortenberg, Germany). Antibody levels were determined from a standard curve, derived by serial dilution of WHO international standards for anti-SARS-CoV-2 immunoglobulin (NIBSC, UK) which was assigned an arbitrary unitage of 1000 BAU/mL. The theoretical curve for single antibody and antigen, and least-squares fit to logit-transformed data model was used to calculate the arbitrary units of samples as described previously^36^. The values were presented as BAU/mL and the cut-off level of seroconversion was set as ≥ 3 times of standard deviation of the negative test value which was 50.7 BAU/mL.

### Neutralization assay

#### The SARS-CoV-2 NeutraLISA (EUROIMMUN Medizinische Labordiagnostika AG, Lübeck, Germany)^37^

The concept of this test intended to mimic virus-host interaction utilizing recombinant RBD of the SARS-CoV-2 spike protein to detect antibodies that block the RBD binding to the hACE2 receptor by using an established ELISA method. The assay was performed following the manufacturer's instruction. The values identified in this study were presented as percent inhibition and the cut-off level of seroconversion was 35%.

#### The in-house SARS-CoV-2 surrogate virus neutralization test (in-house SARS-CoV-2 sVNT)

In-house SARS-CoV-2 sVNT was modified from the method previously described by Tan CW, et al^23^. In brief, 96 well Maxisorp Immunoplates were coated with 50 µl of 4 μg/mL ACE2 (Genscript, Piscataway, USA) in bicarbonate buffer overnight at 4 °C. After washing, the wells were blocked with 100 µl of 2% bovine serum albumin (PAA Laboratory, Pasching, Austria) in PBS and incubated at 37 °C for 1 h. Sixty microliters of the sera of participants or controls diluted in a ratio of 1:4 were pre-incubated with 60 μL of 1:200 HRP-RBD (Genscript) for 30 min at 37 °C. After discarding blocking buffer, 100 μL of the sera-RBD-HRP mixture were transferred to the plates and incubated at 37 °C for 30 min. After washing, 50 μL of TMB substrate solution was added, followed by 50 μL of 0.2 M sulfuric acid stopping solution after a 30 min incubation period. The absorbance was read at 450 nm using a CLARIOstar® microplate reader. Pooled convalescent sera from patients aged 18–59-years old with a PCR-confirmed Alpha or Delta SARS-CoV-2-variant infection admitted at Maharaj Nakorn Chiang Mai Hospital who had provided written informed consent were used as positive controls. Pooled sera from participants collected before SARS-CoV-2 vaccination were used as negative controls. The values were presented as percent inhibition and the cut-off level of seroconversion was 30%, which determined by the mean % inhibition of individual negative serum plus 3 standard deviations. All tests were performed against wild-type SARS-CoV-2.

## Statistical analysis

### Sample size calculation^38,39^

The sample size was calculated based on the comparisons between (1) S-specific-IgG-test and (2) commercial sVNT, and between (3) RBD-specific-IgG-test and commercial sVNT, which required the largest sample size in this study. Using the expected kappa of greater than 0.8 (e.g. 0.9), and probability of positive results for tests (1) and (3) were 0.75 and 0.75, and probability of seroconversion for test (2) was 0.7, respectively, with a two-sided alpha of 0.05, and a power of 80%, 312 samples were required.

### Data analysis

Demographic data including age, gender, and underlying diseases were described as number (%), mean ± SD, and median (IQR) as appropriate. The correlation between 2 tests with continuous outcomes i.e. (1) in-house ELISA v.s. S-specific-IgG-test, (2) in-house ELISA v.s. RBD-specific-IgG-test, (3) S-specific-IgG-test v.s. RBD-specific-IgG-test were log-transformed and analyzed using Pearson's correlation coefficient (*r*)^40^. The correlation between Pearson's correlation coefficient i.e. (1) in-house ELISA v.s. S-specific-IgG-test, (2) in-house ELISA v.s. RBD-specific-IgG-test, (3) S-specific-IgG-test v.s. RBD-specific-IgG-test, the commercial sVNT v.s. in-house sVNT and was analysed using Spearman's rank correlation coefficient (*r*_s_)^40^. Correlation coefficient reported as estimate values and 95% confidence intervals. Correlation strengths of 0–0.09, 0.10–0.29, 0.30–0.59, 0.60–0.79, 0.80–0.99, 1 were defined as none, poor, fair, moderate, very strong and perfect respectively^40^. A scatterplot was used to describe the direction, form, and strength of correlation. We used quantile regression model for converting from in-house sVNT to the commercial sVNT for and converting from the commercial sVNT to in-house sVNT.

Cohen’s kappa was used to measure agreement between 2 tests with binary outcomes. The kappa values of 0–0.20, 0.21–0.39, 0.40–0.59, 0.60–0.79, 0.80–0.90, > 0.90 were defined as no, minimal, weak, moderate, strong, and almost perfect level of agreement^41^.

To identify the value of binding specific IgG antibody which was correlated with 35%, 80%, 85%, 90%, and 95% inhibition of commercial sVNT, 4 sequential steps were performed. First, the regression of binary outcome on continuous outcome with logistic regression, (2) use of the receiver operating characteristic (ROC) curve to assess efficiency of classification, (3) identification of the cut-off point by Euclidian’s index method for ROC curve, and (4) assessment of the accuracy of cut-off point for sensitivity and specificity using commercial sVNTs as a reference standard^42,43^.

### Ethical declarations

This study was approved by the Faculty of Medicine Chiang Mai University Ethics Committee number 4, approval number 311/2564. All methods were performed in accordance with relevant guidelines and regulations.

## Data Availability

The datasets used and analysed during this study available from the corresponding author on reasonable request.
